# Carl Gustav Jung, Quantum Physics and the Spiritual Mind: A Mystical Vision of the Twenty-First Century

**DOI:** 10.3390/bs3040601

**Published:** 2013-11-13

**Authors:** Diogo Valadas Ponte, Lothar Schäfer

**Affiliations:** 1 Associação AVC (Cerebral Vascular Diseases), 4750-175 Barcelos, Portugal; 2 Physical Chemistry, University of Arkansas, Fayetteville, AR 72701, USA

**Keywords:** Archetypes, Cosmic Consciousness, Mysticism, Non-empirical reality, Potentiality, Quantum physics, Spirituality, Unus Mundus, Virtual states

## Abstract

We describe similarities in the ontology of quantum physics and of Carl Gustav Jung’s psychology. In spite of the fact that physics and psychology are usually considered as unrelated, in the last century, both of these disciplines have led at the same time to revolutionary changes in the Western understanding of the cosmic order, discovering a non-empirical realm of the universe that doesn’t consist of material things but of forms. These forms are real, even though they are invisible, because they have the potential to appear in the empirical world and act in it. We present arguments that force us to believe, that the empirical world is an emanation out of a cosmic realm of potentiality, whose forms can appear as physical structures in the external world and as archetypal concepts in our mind. Accordingly, the evolution of life now appears no longer as a process of the adaptation of species to their environment, but as the adaptation of minds to increasingly complex forms that exist in the cosmic potentiality. The cosmic connection means that the human mind is a mystical mind.

## 1. Introduction

When René Descartes declared that the world consisted of two kinds of material, *i.e.*, thinking substance and extended substance, and when Isaac Newton ([1], p. 400) declared that “God in the beginning formed Matter in solid, massy, hard, impenetrable, moveable Particles...so very hard, as never to wear or break in pieces”, Western Science then became a form of materialism, and anything that wasn’t matter didn’t matter. When Darwin introduced Newton’s materialism into biology, having-or-not-having stuff became the essence of life, and greed and aggression became the natural virtues of our society, segregating one individual from the next, one country from another, and one species from the next. In this way, the classical world was a segregative world, and all aspects of life were affected: The physical sciences had nothing to do with ethics, philosophy had nothing to do with the arts, and the order of the universe had nothing to do with the way in which we should live. As Jacques Monod described it: “Man must at last wake out of his millenary dream and discover his total solitude, his fundamental isolation. He must realize that, like a gypsy, he lives on the boundary of an alien world; a world that is deaf to his music, and as indifferent to his hopes as it is to his suffering or his crimes” ([2], p. 160).

In this totalitarian materialistic environment, Carl Gustav Jung had the courage to propose that our mind is guided by a system of forms, the archetypes, which are powerful, even though they don’t carry any mass or energy, and which are real, even though they are invisible. The archetypes exist, as Jung ([3], pp. 43v44) described, in a “psychic system of a collective, universal, and impersonal nature”. Out of this system, the invisible forms can appear in our mind and guide “our imagination, perception, and thinking”.

As it turns out, Carl Gustav Jung’s revolutionary views of the human mind are in perfect agreement with the discoveries of Quantum Physics, which, during the last century, also came as a shock, because they revealed the fundamental errors of Classical Physics and led to a radical change in the Western view of the world. The quantum phenomena now force us to think that the basis of the material world is non-material, and that there is a realm of the world that we can’t see, because it doesn’t consist of material things, but of non-material forms. These forms are real, even though they are invisible, because they have the potential to appear in the empirical world and to act on us. They form a realm of potentiality in the physical reality, and all empirical things are emanations out of this realm. There are indications that the forms in the cosmic potentiality are patterns of information, thought-like, and that they are hanging together like the thoughts in our mind. Accordingly, the world now appears to us as an undivided wholeness, in which all things and people are interconnected and consciousness is a cosmic property. 

In this essay, we will describe the similarities between Carl Gustav Jung’s psychology and Quantum ontology. Our description will show that Jung’s teaching is more than psychology: it is a form of spirituality. By “spirituality”, we mean a view of the world that accepts the numinous at the foundation of the cosmic order. In the same way, Quantum Physics is more than physics: it is a new form of mysticism, which suggests the interconnectedness of all things and beings and the connection of our minds with a cosmic mind.

## 2. Quantum Physics and the Spiritual Foundation of the Empirical World

If we want to characterize Carl-Gustav Jung’s psychology in one sentence, we can say that Analytical Psychology, embodied in the archetype structure, leads us to the view that there is a part of the world that we can’t see, a realm of reality that doesn’t consist of material things but of non-material forms. These forms are real even though they are invisible, because they have the potential to appear in our mind and act in it. In the following sections, we will show that this view of the world is identical with the ontology of Quantum Physics. Our description is necessarily short, but the interested reader will find many details and references in our previous works [4,5,6,7,8,9,10,11,12,13,14,15,16,17,18,19,20,21,22]; particularly, in a recent book, “*Infinite Potential. What Quantum Physics Reveals About How We Should Live*” [23].

## 3. The Basis of the Material World is Non-Material

The first aspect of the quantum world that we have to consider concerns the fact that the basis of material things is not material. This view is in complete contrast to our experience of the world, but it follows from Schrödinger’s quantum mechanics, which is currently the only theory that allows us to understand the properties of atoms and molecules. In this theory, the electrons in atoms and molecules aren’t tiny material particles, little balls of matter, but standing waves or forms. 

All atoms consist of a positively charged nucleus, which contains most of the mass of an atom, and of electrons, which are somehow arranged in the space surrounding the nucleus. Electrons are tiny elementary particles: they have a definite mass and, whenever we see one, it appears as a tiny dot: for example, as a flash on a TV screen or a little mark on a photographic film.

In contrast to their appearances, the electrons in atoms and molecules aren’t tiny material particles or little balls, which run around atomic the atomic nuclei like planets around the sun, but they are standing waves: when an electron enters an atom, it ceases to be a material particle and becomes a wave. We owe Max Born for the discovery that the nature of these waves is that of probability waves. That is, the electrons in atoms are probability fields. 

When this aspect of electrons first became known was unclear. What are probabilities? Probabilities are dimensionless numbers, ratios of numbers. Probability waves are empty and carry no mass or energy, just information on numerical relations. Nevertheless, the visible order of the world is determined by the interference of these waves. The interferences of atomic wave patterns, for example, determine what kind of molecules can form. In addition, the interferences of molecular wave forms determine how molecules interact. The molecules in your body, for example, interact in such a way that they keep you alive. 

In view of these properties of the elementary units of matter, we have to conclude that the order of the visible world is based on phenomena, which transcend the materialism of classical physics. If one pursues the nature of matter to its roots, at the level of atoms and molecules all of a sudden one finds oneself in a realm of mathematical forms and numbers, where all matter is lost: Thus, one is led to the view that the basis of reality is nonmaterial.

In modern science, this finding was unexpected, and many scientists still don’t accept it, but the idea isn’t new. For example, in the sixth century B.C.E. Pythagoras ([24], p. 54) was already teaching that “all things are numbers” and that “the entire cosmos is harmony and number.” In Plato’s philosophy, atoms are mathematical forms. St. Augustine wrote in his *Confessions*: “The older I got, the more despicable became the emptiness of my thought, because I could think of no entity in any other way than as bodily visible”. Moreover, Nicolas da Cusa, a fifteenth century German theologian, is credited with the statement: “Number was the first model of things in the mind of the Creator.”

At this point, the reader may already note the importance of the quantum world for Carl Gustav Jung’s psychology: The discovery of a realm of non-material forms, which exist in the physical reality as the basis of the visible world, makes it possible to accept the view that the archetypes are truly existing, real forms, which can appear in our mind out of a cosmic realm, in which they are stored. Thus, we can confirm here on the basis of the quantum phenomena Jung’s view that “it is not only possible but fairly probable, even, that psyche and matter are two different aspects of one and the same thing” ([25], para. 418). 

## 4. Consciousness Is a Cosmic Property

An important concept that arises in the Quantum phenomena concerns the wholeness of the physical reality. By the concept of wholeness, we mean that seemingly separated things can be connected and can act instantaneously on each other over arbitrarily long distances. In a holistic universe, decisions made by an observer in one part of the world can have an instantaneous effect on the outcome of processes somewhere else, an arbitrarily long distance away. For example, a thought that appears in my mind at this moment may instantly appear in your thinking somewhere else, in another part of the world. In physics, we speak of “nonlocality”, when two particles, which at one time interact and then move away from one another, can stay connected and act as though they were one thing, no matter how far apart they are.

In the world of ordinary things, no influence or signal can travel at a speed faster than the speed of light. Thus, any action taken at one part of the world can have an effect somewhere else only after the time that it takes for a signal to get from one point to the other. In the quantum world, the situation is different: Influences can act instantaneously over arbitrarily long distances; in principle, from one end of the universe to another.

The aspect of the wholeness of reality can be described in a simple way in connection with the wave properties of elementary particles. In the previous section, we have seen how the electrons in atoms are waves. Whenever we see an electron, it appears as a material particle. However, inside an atom, it is a wave.

This metamorphosis of particles to waves and waves to particles is a general phenomenon that doesn’t only describe the modes of existence of electrons, but is a characteristic of all elementary particles, atoms and molecules. It means that, whenever we see what we call an elementary particle, it appears as a tiny material thing at a specific position in space. In contrast, when such a thing is on its own, like when it is in a vacuum, it ceases to be a material particle and becomes a wave. You can think of this process as a spontaneous transition of what we see as a particle from its particle state to a wave state. 

In “*Infinite Potential*” [23] this phenomenon has been described in the following way: At the foundation of the visible world we find Entities, which always appear to us as Elementary Things, when we interact with them. However, when they are on their own, they become waves. As waves, they have lost all mass, and they have become pure forms, patterns of information, something mindlike or thoughtlike. Accordingly, we can call the units of existence at the foundation of the world “ETs”, meaning *E*lementary *T*hings, of *E*lementary *T*houghts; or, simply, *E*nti*Ties*. 

Being a localized material particle is one state of existence of an ET; being a non-material wave is another. As it turns out, the wave state is the preferred state of an ET: It is the home, where it will go, when it is left alone. As a wave, an ET has lost all of its mass. It has become a nonmaterial and invisible form and, since waves are extended in space, it has no specific position in space, but many potential positions. We say that an ET in its wave state is in a *state of potentiality*. Since material particles, whenever we see one, always appear with a specific mass at a specific point in space, we must conclude that ETs in a state of potentiality aren’t a part of the empirical world. By making a transition into a wave state, an ET leaves the empirical world.

This phenomenon is general and cosmic: There is a realm of the universe that we can’t see. It is a background of nonmaterial forms, not things. The forms are real, even though they are invisible, because they have the potential to appear in the empirical world and act in it. In fact, we must now think that the entire visible world is an emanation out of a non-empirical cosmic background, which is the primary reality, while the emanated world is secondary.

We can’t really know what the nature of the ETs is in the non-empirical background of the world. Indications are that they have wavelike properties. If so, we must think that the background of the visible world is like an ocean. The ETs in this ocean are hanging together, like the water waves in an ocean do, so that the nature of reality is that of an indivisible wholeness. 

The wholeness of the cosmic background is also suggested by the following consideration: If the ETs in the realm of potentiality wouldn’t form a coherent whole, the empirical world that is emanating out of the cosmic potentiality would be chaotic. However, the visible isn’t chaotic. Rather, it always appears to us as a coherent system.

As patterns of information, the ETs in the realm of potentiality are more thoughtlike than thinglike. Thoughts usually appear in a conscious mind. Thus, the appearance of thoughtlike forms in the cosmic potentiality suggests that consciousness is a cosmic property. The universe is conscious and our thinking is the thinking of the cosmic mind, which finds consciousness in us!

The same conclusions follow from the holistic nature of reality. For example, in their book, “*The Conscious Universe”*, Menas Kafatos and Robert Nadeau [26] have argued that, if the universe is an indivisible wholeness, everything comes out of this wholeness and everything belongs to it, including our own consciousness. Thus, consciousness is a cosmic property. 

This quantum view of a holistic reality is in perfect agreement with one of Jung’s most important seminal ideas; that is, the archetypal idea of *Unus Mundus*, which Jung [27] and Marie-Louise von Franz [28] derived from characteristic medieval views of the world. In Jung’s words:

“Undoubtedly the idea of the Unus Mundus is founded on the assumption that the multiplicity of the empirical world rests on an underlying unity, and that not two or more fundamentally different worlds exist side by side or are mingled with one another. Rather, everything divided and different belongs to one and the same world, which is not the world of sense.”.

([27], para. 767)

Ontologically, this archetype means that there is a reality that must be united, “apparently” divided, opposed, but beyond the illusion of matter, it is One. The reader will note the agreement of Jung’s views with the quantum view of the world that we have described above. 

The process of individuation is an innate capacity of the individual to become aware of the Self. According to Robert K. C. Forman [29], we have an innate capacity, which is an imperative, long life process of transformation. This is an impulse to unite what is divided. In “*The Archetypes and the Collective Unconscious*” Jung affirms that “I use the term ‘individuation’ to denote the process by which a person becomes a psychological ‘in-dividual’, that is, a separate, indivisible unity or ‘whole’” ([3], p. 275). Searching for wholeness would be meaningless in a Newtonian world of separate material things. In the quantum world, it has found a physical basis. 

Jung also understood the process of individuation as a religious impulse, which is a wholesome spiritual archetype that directs and coordinates the flow of human life. The word religious is used in this context in the sense of its etymological roots, in which *Re-Ligare* means “to reconnect,” or “to be in bond,” or “to re-unite”. As Anniela Jaffé wrote:

“Individuation must be understood in religious language as the realization of the ‘godly’ in the human, as the fulfilling of a ‘godly mission’. The conscious experience of life becomes a religious experience, one could just as well say, a mystical experience.”.

([30], pp. 14–15)

Another characteristic aspect of Jung’s work is his fascination with Alchemy [31] and, specifically, with the Philosophers’ stone as a metaphor of the process of individuation. Jung considered this process as a transformational journey into the wholeness, in which we bring the invisible to the visible, spiritualize matter and materialize the spiritual. In “Septem Sermones ad Mortuos” (The Seven Sermons to the Dead, re-published in the recent Red Book, Jung, [32]), he uses the Gnostic term “Pleroma” to refer to the wholeness.

In agreement with the aspects of wholeness that appear in the quantum view of the universe, Jung believed that the psyche has a natural and innate urge toward wholeness. Henderson has pointed out that

“a sense of completeness is achieved through a union of the consciousness with the unconscious contents of the mind. Out of this union arises what Jung called ‘the transcendent function of the psyche’, by which a man can achieve his highest goal: the full realization of the potential of his individual Self.”.

([33], p. 149)

The craving for the wholeness is the real “opus” that underlies all of Jung’s work. In accordance with quantum physics, the meaning and purpose of our nature is anchored in the numinous realm of reality. As Jung describes the spiritual quest:

“The main interest of my work is not concerned with the treatment of neurosis, but rather with the approach to the numinous. But the fact is that the approach to the numinous is the real therapy, and inasmuch as you attain to the numinous experience, you are released from the curse of pathology. Even the very disease takes on a numinous character.”.

(Jung cit. in [30], p. 16)

As we have pointed out before [21,22] the path of Ethos needs a non-empirical domain of reality. This invisible realm, which Jung assumed as “psychoid”, provides an infinite field for the progress of the Ego-Self axis relation, nurturing consciousness as an element in which every phenomenon collapses. Quantum physics brings us a new kind of reality, in which it is our task to unlock our potential and to free us from our ignorance, the biggest shadow of all. In agreement with Jung’s analytical psychology, Quantum physics provides us with direct suggestions of how we can live in accordance with the numinous realm of the universe.

Joseph Campbell [34] has used the metaphor of the hero to describe the process in which the Ego unites with the self. In the first half of our life, our Ego is separated from our unconscious. However, after this period, it has a longing to reach a primordial state of wholeness, facing all kinds of dangers and trials. The Portuguese language has a specific word for this longing: that is, *saudade.* We find this myth in countless ancient spiritual teachings (cf. [34]), in the writings of the classical poets, and now it reappears in the worldview of quantum physics. Anniela Jaffé writes:

“in religious language an image of a God who seeks man just as much He is sought by man. God seeks the individual in order to realize himself in his soul and his life. Expressed psychologically: the Self requires the ego-personality in order to manifest itself; the ego-personality requires the Self as the origin of its life and its fate. In religious language this means ‘God needs man, just as man needs God’.”.

([30], pp. 17–18)

As Jung wrote to Erich Neumann: “God is a contradiction in terms, therefore he needs man in order to be made One…God is an ailment man has to cure.” ([30], p. 99).

## 5. Eddington’s Views of a Conscious Universe

In the 1930s, Sir Arthur Stanley Eddington a prominent British astrophysicist, was one of the first physicists who systematically searched for aspects of consciousness in the universe, concluding that “The universe is of the nature of ‘a thought or sensation in a universal Mind’” ([35], p. 151).

One of Eddington’s arguments was based on the fact that, when physicists make measurements, their observations make sense, because the measuring instruments are connected with a meaningful background of the objects that are measured. For example, when we observe the movement of a light dot through the sky at night, our observations make sense because we know the planetary background, where the planets revolve about the sun. In this situation, Eddington pointed out, observations of atoms are a problem, because their background isn’t known. Whenever we see an atom, we can see phenomena that occur at its surface, but we don’t know, what happens inside. Why is the background of atoms not known and even unknowable? Because, for example, as we have described above, the electrons in atoms are nonmaterial, nonempirical forms, and we don’t know what that means. “Now we realize”, Eddington ([36], p. 259) wondered, “that science has nothing to say as to the intrinsic nature of the atom”.

If science has nothing to say about the building blocks of the visible world, it is a problem that must be addressed. As it turns out, it isn’t the only puzzle of its kind. A similar situation arises, for example, in neurology, where no measurements of the surface of a brain can tell us what is going on in the mind behind it.

In spite of this similarity, watching a brain is fundamentally different from watching an atom. This is so, because behind the surface of a brain there is a mind and a person, who can tell, what is going on in this mind. In contrast, atoms aren’t connected with elementary persons who live inside and can tell us what is going on behind the surface. Nevertheless, Eddington suggested thinking of the two situations together, that of the brain and that of the atom, and he concluded that the background of atoms is mindlike. Since we need something to which we can attach the measurements of an atom,

“why not then attach it to something of spiritual nature of which a prominent characteristic is thought. It seems rather silly to prefer to attach it to something of a so-called ‘concrete’ nature inconsistent with thought, and then to wonder where the thought comes from”.

([36], p. 259)

The last part of this statement is a surprise: we usually take our thinking for granted, and the thoughts in our mind tell us a lot of things, but they say nothing about where they are coming from! Is our mind an invention of our brain? Or, do we have a mind because the background of the universe is mindlike and expresses itself in our mind? To Eddington the “unity” of the universe made it necessary to conclude that, behind all empirical appearances of the world, “there is a background continuous with the background of the brain” ([36], p. 312). Unity in this context means coherence. That the universe is a coherent system can be suggested on the basis of the unity of our mind: “If the unity of a man’s consciousness is not an illusion, there must be some corresponding unity in the relations of the mind-stuff, which is behind [the visible surface of things]” ([36], p. 315). Thus, from our inner sense of unity we infer the unity of the world. If the universe wasn’t a coherent system, but a random collection of disconnected piles of material debris, the unity of our thinking would be an illusion. On the other hand, if the universe is a coherent whole, the existence of our personal mind suggests that the background of the universe is mindlike. 

In this way, Eddington was lead to the conclusion that,

“The universe is of the nature of ‘a thought or sensation in a universal Mind’...To put the conclusions crudely—the stuff of the world is mind-stuff. As is often the way with crude statements, I shall have to explain that by ‘mind’ I do not here exactly mean mind and by ‘stuff’ I do not at all mean stuff. Still this is as near as we can get to the idea in a simple phrase”.

([36], pp. 259–260)

Eddington ([36], p. 281) realized that his views were alien to physics. “It is difficult for the matter-of-fact physicist to accept the view that the substratum of everything is of mental character.” 

However, this is a problem of physics, not of Eddington’s theses, and it shows the inability of the physical sciences to describe all the essential aspects of the universe.

Even though they are controversial, Eddington’s theses are in perfect agreement with Carl Gustav Jung’s basic assumptions, and with the quantum phenomena, which show us that there is a part of the world that we can’t see, a background of potentiality, that doesn’t consist of things, but of forms. These forms are thought-like, not thing-like, and they are real because they can actualize in the empirical world and act in it. As a matter of fact, the entire empirical world now appears to as an emanation out of a realm of invisible forms.

The agreement, if not identity, with Jung’s basic theses is striking: our conscious thinking is based on an emanation of forms out of a non-personal, that is, cosmic realm. 

“Consciousness is not sharply defined”, Eddington [36] explained, “but fades into subconsciousness; and beyond that we must postulate something indefinite but yet continuous with our mental nature. This I take to be the world-stuff”. We compare the mind-stuff “to our conscious feelings,” Eddington concluded, “because, now that we are convinced of the formal and symbolic character of the entities of physics, there is nothing else to liken it to” ([36], p. 280).

## 6. Quantum Physics Is the Psychology of the Universe

An important concept in quantum chemistry is the concept of virtual states: virtual states are the empty states of atoms and molecules. (For a more detailed description of the concept of virtuality in chemistry, with additional examples, see “*Infinite Potential*” [23].

All atoms and molecules exist in quantum states. You can think of a molecule like of a mountain range with countless hills and valleys. Each valley is an energy hole, which contains an energy ladder. The steps of these ladders represent fixed, or quantized, amounts of energy: they are the quantum states of a molecule. Each molecule must occupy one of its states—it must stand on one of the steps of its ladders—so that a large number of states are empty. Quantum chemists call the empty states of things their *virtual states*. Virtual states are mathematical forms or patterns of information. They have the forms of waves, but these waves are invisible, because they are empty: there is nothing there to see. But they are real and they truly exist, even though we can’t see them, because a molecule can jump into such a state and make it a visible state. You can think of virtual states as the logical structure of a system, which contains its future empirical possibilities: All that a molecule can do is to jump from an occupied state into a virtual state.

In an empirical science the appearance of entities, which have no matter, no energy and are invisible, is an embarrassment. You can very well compare the situation to Jung’s thesis that behind our conscious thinking there is a realm of unconscious forms. If you have to describe the world by referring to an invisible, numinous realm of reality, you are leaving the realm of empirical science. Thus, many of the pioneers of quantum physics tried to explain the virtual states away as mere constructs that don’t really exist. However, we have no choice: we have to think that the empty states of atoms and molecules are real, because they can control empirical phenomena. 

For example, all chemical reactions are steered by the virtual states of the reacting molecules, which determine what kinds of molecules can form in a reaction. In a specific type of reactions, called Redox reactions, the products appear with characteristic magnetic properties, which are determined by their virtual states. In addition, oxygen can serve our metabolism, because it contains what chemists call degenerate states. Degenerate states are invisible and yet they are the basis for the particular reactivity of oxygen. 

There is no doubt: invisible virtual states are real. Since their inner forms can affect visible phenomena, they must be truly existing, real entities. Molecules are guided in their actions by the wave forms of their virtual states, like by inner images. 

The concept of the inner images derives from psychology. Brain scientist Gerald Hüther ([37], p. 17) calls inner images all that “which is hidden” behind the visible surface of living beings and steers their actions. Similarly, Jung [3] believed that archetypal images exist in our consciousness, which are manifestations of the pure forms of archetypes, which are unknowable.

In chemistry, a molecule doesn’t do anything that isn’t allowed by a wave form—an inner image—of one of its virtual states. In life, a human being does nothing that isn’t allowed by an inner image of the mind. There is an equivalence of the mental and the physical. Psychology is the physics of the mind: Quantum physics is the psychology of the universe. 

## 7. Quantum Wave Functions Are Archetypes

It is no accident that the development of psychology as a science took a quantum leap after 1900 C.E, when the era of the Classical Sciences came to an end and the Quantum era began. Jung’s view of the human psyche presupposes a structure of the universe that is in perfect agreement with the Quantum universe, but impossible in Newton’s world. For example, Jung’s assumption that an invisible part of the world exists, which doesn’t consist of material things, but of forms—the archetypes—is unacceptable in a Newtonian universe, in which all phenomena depend on the properties of matter. 

Jung’s *collective unconscious* is a non-personal part of the human psyche. It is a realm of forms—*the archetypes*—which can appear spontaneously in our consciousness and act in it, influencing “our imagination, perception, and thinking” ([3], p. 44). The archetypes are “typical modes of apprehension” ([25], p. 137), which shape, regulate and motivate the conscious forms in our mind in the same way, in which the virtual states of atoms and molecules shape and control empirical phenomena. We must constantly reach into the realm of the archetypes and actualize their virtual forms, in order to be able to live and to give meaning to life. 

We have described above, how molecules are guided in their actions by the wave forms of their quantum states, like by inner images. Since the inner images control all the processes of the world, they must have guided, too, the evolution of life. In this way, biological evolution appears primarily not as an adaptation of life forms to their environment, but as the adaptation of minds to increasingly complex forms—archetypes—in the cosmic potentiality. In our minds, the cosmic forms appear as thoughts; in the physical reality they appear as material structures. We can understand the world, because the forms within our mind and the structures of the world outside, both derive from the same cosmic source.

It makes sense to think that all of reality is like the reality of the atoms. That is, behind the visible surface of things there is a realm of invisible forms, which have the potential to appear in the empirical world and act in it. As pointed out above, we can think of this realm like of an ocean, whose waves are hanging together and are mind-like, so that the universe now appears as an indivisible wholeness, and consciousness is a cosmic property. 

The appearance of the archetypes in our mind shows our connection with a transpersonal order. Beyond the narrow confines of our personal psyche, Jung pointed out, the collective unconscious is

“a boundless expanse full of unprecedented uncertainty, with apparently no inside and no outside, no above and no below, no here and no there, no mine and no thine, no good and no bad…where I am indivisibly this and that; where I experience the other in myself and the other-than-myself experiences me…There I am utterly one with the world, so much a part of it that I forget all too easily who I really am.”.

([3], p. 21)

Idealist philosophers and mystics have pursued such ideas through the ages. In the nineteenth century, for example, Georg Wilhelm Friedrich Hegel taught that “Absolute Spirit” is the primary structure of the universe. Everything that exists is the actualization of spirit, and everything is connected with it. Spirit is everything, creates everything, and thinking and being, subject and object, the real and the ideal, the human and the divine—all are One. Thus, Hegel concluded, our thinking is the thinking of the Cosmic Spirit, who is thinking in us.

Thousands of years prior to Hegel, the Indian Sages invented the allegory of the water pots, which are filled with water and placed into the sun: You can see the sun in each one of them, but there is only one sun. Similarly, you can find consciousness in countless human minds, but there is only one consciousness: the Cosmic Consciousness.

The word, “consciousness” derives from the Latin, “con” and “sciencia”, and it means a state of “knowing together”. Interestingly, when we speak of our consciousness and that of other people, we always speak of “our consciousness”, and never use the plural form, speaking of our consciousnesses. There is no plural form, because there is only one consciousness: the cosmic consciousness. If our personal consciousness is merely a part of a cosmic system, it isn’t amazing that archetypes can appear in our mind and act in it. 

By the way, in which it describes the world, quantum physics has taken science into the center of ancient spiritual teachings. For example, molecular wave functions have no units of matter or energy. They are pure, non-material forms. The same is true for Jung’s archetypes: like the wave functions of quantum systems, they are pure, non-material forms. In Aristotle’s metaphysics, all things are mixtures of matter and form. There was only one pure form: God. 

The name that quantum chemists have given the empty states of atoms and molecules—that is, calling them “virtual states”—is a peculiar expression and one wonders, where it is coming from? As it turns out, the concept wasn’t invented by quantum chemists, but by Meister Eckhart, a medieval Dominican Monk and Mystic. “The visible things are out of the oneness of the divine light”, Meister Eckhart (cit. in [38], pp. 63–64) wrote, and their existence in the empirical world is due the “actualization of their ‘virtual being’”.

What a stunning phenomenon! The same unusual term appears in the mind of a medieval mystic and then, hundreds of years later, in the mind of a quantum chemist. The example shows, that absolute truths can appear, again and again, with the same messages, through thousands of years, in different minds, different ages and different parts of the world. It is difficult to avoid the impression that our minds are connected to a cosmic realm of thoughts: the realm of Jung’s archetypes.

Jung’s archetypes and the wave functions of quantum states are so similar that we could think of the archetypes as the virtual state functions of our mind; and we could speak of the virtual quantum wave functions as the archetypes of the physical reality. Because they “have never been in consciousness” before ([3], p. 42), the archetypes appear out of a nonempirical realm of the world. For each one of us the birth of a conscious self is out of a realm of nonempirical forms, in the same way in which the birth of an empirical world is out of a realm of virtual states. It is difficult to avoid the conclusion that the two families of forms have their home in the same cosmic realm; that is, in the realm of the cosmic consciousness. “That the world inside and outside ourselves rests on a transcendent background is as certain as our own existence.” (Jung cit. in [30], p. 4).

## 8. Synchronicity and the Mindlike Background of the Universe

Carl-Gustav Jung is primarily recognized as a revolutionary psychiatrist and psychotherapist. However, the aspects of the psyche that he discovered are so profound, that they go beyond the limited concerns of the human psyche, making it possible to think, for example, that the universe itself is conscious and our own consciousness is connected with the cosmic consciousness.

In Jung’s theories, the concept of synchronicity plays an important role. Jung’s German term, sinngemäße Koinzidenz, means a “coincidence according to meaning”. It is usually translated as “meaningful coincidence”, referring to the coincidence of two or more events, and it describes phenomena in which an event in the external world coincides meaningfully with a psychological state of the mind; that is, two or more events are connected in meaning but not in their visible causes. As Jung ([25], para. 858) describes it, in the simultaneous appearance of synchronistic events “something other than the probability of chance is involved”. Specifically, synchronicity “consists of two factors: (a) A unconscious image comes into consciousness either directly (*i.e*., literally) or indirectly (symbolized or suggested) in the form of a dream, idea, or premonition. (b) An objective situation coincides with this content. The one is as puzzling as the other.”

When someone dreams of an unusual event, and the next day that same event actually happens in another part of the world, then we are dealing with a case of synchronicity. As Jung ([25], pp. 520–531) pointed out, such experiences are particularly stunning, when an inner mental state coincides with an external event that “takes place outside the observer’s field of perception, *i.e.*, at a distance, and only verifiable afterward”.

In the framework of classical physics, coincidences according to meaning are impossible as non-random events. That is, classical physics doesn’t allow causally connected, physical phenomena, which don’t involve the exchange of physical energy or forces. Jung ([25], pp. 520–531) was aware of this problem. “No one has yet succeeded”, he wrote, “in constructing a causal bridge between the elements making up a synchronistic coincidence”. Nevertheless, he had no doubt that synchronicity was a real phenomenon that is “based on some kind of principle, or on some property of the empirical world”. The quantum phenomena make it now possible to identify this property. However, as it turns out, it isn’t a property of the empirical world, but it involves the non-empirical realm of reality.

We have seen above that the phenomena of quantum physics force us to conclude that reality appears to us in two domains. There is the domain of the empirical, energetic and material things: the realm of the actuality of the visible phenomena. However, in addition, behind the visible surface of things is a hidden, invisible and non-empirical domain that doesn’t consist of things, but of nonmaterial and nonempirical forms: the realm of the potentiality of the universe. You could think that the visible world is something like the consciousness of the universe; while the hidden part is its unconscious.

We have said that the nonempirical forms in the cosmic realm of potentiality are real, because they have the potential to appear in the empirical world and act in it. They can do this in two ways. They can appear as thoughts and images in our conscious mind; and as material structures and events in the external world. When one and the same form appears, at the same time, both as a thought and as an external event, a mental process and an empirical occurrence express the same meaning, and we experience a synchronistic event. In a Newtonian world, such events are impossible; in a quantum world, they *must* occur. We can’t know what causes such events, because their causes, if any, are nonempirical. However, we can understand that synchronistic events are possible, because the universe is an indivisible wholeness that is aware of its processes, like a Cosmic Spirit. Thus, we are led again to Hegel’s thesis that a Cosmic Spirit is thinking in us.

The lack of visible causal connections is an interesting aspect of synchronistic events. However, in the same way in which quantum events seem random, but are really caused by some nonempirical processes, so the randomness of synchronistic events is only an apparent randomness. The cosmic spirit is unfathomable, but not arbitrary or mindless.

Synchronicity can involve more than a single mind and more than a few events. In the early 1900s, for example, Europe went through an era of revolutionary changes, which affected all aspects of life and show all the characteristics of synchronistic events. In 1900, for example, Sigmund Freud invented psychoanalysis, and Max Planck founded quantum physics. In 1903, Henry Ford founded the Ford Motor Company, and the Wright Brothers succeeded in the first human motor flight. In 1905, Albert Einstein developed Relativity Theory, and in Paris the first modern art show presented paintings by André Derain and Henri Matisse. In 1907, Cubism was developed by Georges Braque and Pablo Picasso. In 1910, Arnold Schönberg wrote the first composition of atonal music. In 1912, Wassily Kandinsky invented abstract painting. In 1913, Franz Kafka published his short stories. In 1914, James Joyce wrote The Dubliners and the First World War began, and 1917 was the year of the Russian Revolution. 

All of these developments were revolutions in their corresponding fields. We perceive a synchronistic connection between these revolutions, because they had a common meaning: that is, each one of them took a given field away from the visible surface of things into a hidden, abstract and more fundamental realm of the world. For example, when quantum physicists discovered the nonempirical realm of the world, the painters of Modern Art began to search for the essence of things behind their visible surface; and psychologists discovered the hidden power of the unconscious. As Werner Haftmann [39] explains in his fascinating book Painting in the 20th Century, paintings became “evocative” and stopped being “reproductive”. When physicists abandoned the notion of the eternal point like particle in quantum physics, the visual artists abandoned, in abstract paintings, the infinite point of perspective, which was the cornerstone of all classical paintings. In “*Infinite Potential*” [23], the reader can find additional facts, which show in a stunning way that the cultural and political revolutions that rocked Europe in the early twentieth century consist of a sequence of synchronistic events. 

There was little physical contact or direct communication between the various pioneers of that time. The physicists, for example, didn’t invent the phenomena of quantum physics by pondering the paintings of modern artists. Modern art wasn’t invented by artists, while they listened to atonal music. Rather, the different minds were connected in the wholeness of the mindlike background of the cosmic potentiality: The cosmic spirit was at work in a synchronistic process.

By guiding the processes of our mind, the cosmic potentiality has shown its mindlike properties. The mental isn’t fractured in the universe in isolated islands, but its thoughts form an ocean of thoughts that fills the entire world.

## 9. Conclusions

By studying the human psyche, Jung discovered mental properties of the universe, which Classical physics had suppressed: Quantum physics has now brought them back.

“If Materialism is false”, writes Imants Baruss ([40], p. 41), “then what is true?” In “*Infinite Potential*” we have answered this question in many ways [23]. The facts show us that there is a non-empirical realm of reality, that doesn’t consist of things, but of forms. These forms are real, even though they are invisible, because they have the potential to appear in the empirical world and act in it. They can do this in two ways: they can find consciousness as thoughts in our mind; and actualize as material structures in the external world. Thus, the conscious and empirical world is an emanation out of a realm of mind-like forms, and quantum physics is a form of psychology, the psychology of the cosmic mind. In the same way Jung’s psychology is also a branch of physics; that is, the physics of the mental order of the universe.

A holistic universe is necessarily a mystical system. Scientific theories, which claim that all things and people are interconnected in a non-empirical realm of the world, are necessarily mystical theories. Jaffé has described the same conclusion in the following way:

“Both Junguian psychology and mysticism deal with the experience of the numinous. The difference is that mysticism speaks of an encounter with God and lets the matter test at that. Jungian psychology also speaks of an encounter with God, in the sense that ‘God’ represents the word or the designation for something incognizable and incomprehensible. For both God is a primordial human experience…”.

([30], p. 12)

Thus, Quantum physics is a form of mysticism; and so is Jung’s psychology. One hesitates to express such conclusions, but the form of mysticism that we find in contemporary science is different from its historic forms. This is so, because our concepts evolve in the same way in which our bodies evolve. 

The evolution of our thinking is characterized by the fact that there are truths regarding the order of the world, which are so fundamental that they have appeared again and again, in the minds of different people, in different ages and in different parts of the world. The Indian sages called this phenomenon Sanatana Dharma. In the sixteenth century, Agostino Steuco, an Italian humanist, introduced the concept into Western philosophy as “perennial philosophy”. We consider this phenomenon as a special form of synchronicity. It shows that our mind is a mystical mind, because it is connected with a cosmic background that has mindlike properties: That is, a cosmic mind.

For some reason, in our history, worldviews have always been accompanied by threats. That is, if you didn’t believe a certain story of how the world was created by God, you were threatened to go to hell, and it isn’t too long ago, that dissidents were really put on fire. Similarly, a scientist may find herself fired out of her job in no time, when she dares to question the narrow mind frame of the contemporary sciences!

Ancient concepts of the world are constantly reemerging in our thinking, but they are doing this in an evolving way. For example, Plato’s claim, that true reality resides in a realm of ideas outside of the visible world, is very similar to the claim that the empirical world actualizes out of a realm of virtual quantum forms. Nevertheless, the quantum view isn’t identical with the Platonic view. Rather, it is mathematical, quantitative and it has led to countless practical applications that have changed our way of life. In the same way, Jung’s descriptions of the human psyche may be similar to ancient views, but they are evolved versions of ancient views. We believe that the evolution of concepts and their understanding is the true function of biological evolution. It is impossible to know, whether we are evolving with the cosmic mind, or whether it is merely our mind that has to evolve to a better understanding of a non-evolving cosmic order. 

The practice of mysticism is an example of an evolving process. When we say that Quantum physics and Jung’s psychology are modern forms of mysticism, we don’t mean that they are identical with ancient religious practices. Rather, they share essential aspects with ancient practices in an evolved way. In her appealing book, “*Was C. G. Jung a Mystic?*”, Aniela Jaffé [30] has described fascinating aspects of Jung’s mysticism, which confirm our view:

“If the concept ‘mystic’ suggests the immediate experience of the numinous or the perceiving of an originally hidden transcendent reality, the ‘other side’, then it involves an experience which also plays a central role in Jung’s approach to analytical psychology; that is, the consideration of images and contents which enter into consciousness from the hidden background of the psyche, the collective unconscious. (…) [which] must be conceived of as a realm with neither space nor time that eludes any objective knowledge. What we perceive are its effects.”.

([30], pp. 1–2)

At this point of our analysis, we might ask: Does it all matter? Why should we care? Our answer is the belief that happiness in this life can be found only by understanding the spiritual background of the universe, and by living in accordance with it. Carl Gustav Jung has shown that, living in accordance with the order of the universe is a prerequisite for a wholesome life. This means that we have to recognize the invisible background of reality and accept the importance of spirit in our life.

As shown in “*Infinite Potential*” [23], the quantum phenomena corroborate Analytical Psychology in the sense that the invisible layer of reality is not only the source but also the goal of our human significance. Influenced by Hindu Advaita philosopher Sankara, Forman has expressed similar ideas:

“One’s atman [wholeness] cannot be ‘produced’ or ‘attained’, for it is already present (…) is the natural condition of the human spirit (…) The activity that seems to bring about the experience of it does so only by destroying the bondage that had hidden it. We are only revealing what had been present all along but hidden: atman. The mystic’s techniques are not ‘producing’ something new but ‘revealing’ something preexistent: ‘Thought Atman is an ever present reality, yet because of ignorance It is unrealized. On the destruction of ignorance, Atman is realized. It is like the case of the ornament on one’s neck.’ Discovering Atman [wholeness] is like finding a necklace hanging on one’s neck: it has always been present and is indeed available, just overlooked. This image emphasizes that atman, and with it the possibility of its realization, is already present to one. It is, in a word, innate.”.

([41], p. 8)

The state of being innate upholds a Cosmic Order that lets us think that we are part of it, that we are born in it and that we are it, but we don’t know it. In agreement with Jung’s Weltanschauung, Quantum physics confirms William James’ thesis, that twenty-first century science can no longer deny the non-empirical:

“[The] unseen region in question is not merely ideal, for it produces effects in this world. When we commune with it, work is actually done upon our finite personality, for we are turned into new men, and consequences in the way of conduct follow in the natural world upon our regenerative charge. But that which produces effects within another reality must be termed a reality itself, so I feel as if we had no philosophical excuse for calling the unseen or mystical world unreal.”.

([42], p. 516)

The view that reality has a non-empirical background can be found at various times in the history of philosophy. We find it, for example, in the theses of the Greek Pythagorean philosopher Timaeus of Locri (420–380 BCE). “God is a circle”, he wrote, “whose center is everywhere and circumference nowhere”.

The main goal of every spiritual tradition is to unite with the transcendent reality. Different traditions may give different names to the divine, but in all of them we find the same desire to become one with the Divine. Psychically, that state can adopt the symbolic and transformational meaning of rebirthing, synonymous with becoming one with the Self:

“To the Indian it is clear that the self as the originating ground of the psyche is not different from God, and that, so far as a man is in the self, he is not only contained in God but actually is God. Shri Ramana is quite explicit on this point. (…) The goal of Eastern religious practice is the same as that of Western mysticism: the shifting of the center of gravity from the ego to the self, from man to God. This means that the ego disappears in the self, and man in God”.

([43], p. 581)

Jung’s teaching is an incredible achievement and a blessing for humanity. He has shown that we are connected with a non-empirical realm of the universe, in which we can find our cosmic task. Denying the transcendent aspects of our nature can lead to serious problems for our physical health and spiritual well being. Our cosmic task isn’t the task of slaves, who have to serve their creator. We are not the slaves of the cosmic spirit, but, rather, we *are* it, if only we try!
